# Involvement of the IFN-gamma pathway in a patient with candle syndrome carrying a novel variant of PSMB8 gene

**DOI:** 10.1186/1546-0096-12-S1-P249

**Published:** 2014-09-17

**Authors:** Antonella Insalaco, Giusi Prencipe, Manuela Pardeo, Virginia Messia, Andrea Masotti, Cristina de Min, Claudia Bracaglia, Rebecca Nicolai, Ivan Caiello, Fabrizio De Benedetti

**Affiliations:** 1 Division of Rheumatology, Department of Pediatric Medicine, IRCCS Ospedale Pediatrico Bambino Gesù, Rome, Italy; 2 Novimmune SA, Geneva, Switzerland

## Introduction

Chronic atypical neutrophilic dermatosis with lipodystrophy and elevated temperature (CANDLE) syndrome is a newly described autoinflammatory disease.

## Objectives

To describe clinical phenotype and cytokine profile of our patient.

## Methods

A 10 year-old young girl presented at 10 months of age with recurrent fever, hepato-splenomegaly and nodular erythematous skin lesions of trunk and limbs; subsequently she progressively developed lypodistrophy, arthralgia, arthritis and edema of eyelids. Skin biopsy showed typical features of lobular panniculitis. Laboratory tests showed persistent elevated acute phase reactants with increased serum amyloid A, persistent chronic anemia, mild recurrent leucopenia, thrombocytopenia and decreased IgA, IgG and IgM levels. Immunological and cytogenetic studies performed on bone marrow were normal. Subsequently, the patient developed proteinuria with nephrotic syndrome. Renal biopsy revealed a minimal change glomerulopathy; she was started on a standard nephrotic syndrome high-dose glucocorticoid protocol with remission of proteinuria. Response to hydroxychlorochine, colchicine, cyclosporine-A, and anakinra was unsatisfactory. Complete sequencing of TNFRSF1A and MVK genes showed no mutations. Analysis of the PSMB8 (proteasome subunit β type 8) gene revealed the presence of c.220A>T p.(T74S) variant in heterozygotic status that has never been reported before. In order to assess the dysregulated inflammatory response in our patient, we evaluated the cytokine profile in patient’s sera using the Luminex multiplexing assay. Whole blood RNA analysis was also performed using a human immune array (TaqMan® Human Immune Array from Applied Biosystems), containing 92 genes typically involved in the immune response.

## Results

Serum samples (n=4) were collected during the last two years. We found high levels of IFN gamma (mean ± S.D.: 115.3 pg/ml ± 64.21), of IFN-gamma inducible protein 10 (IP-10, also called CXCL10) (1641± 892.5 pg/ml) and of IFN-gamma inducible protein 9 (IP-9, also called CXCL11) (582.9± 335.6 pg/ml) and especially of CXCL9, also known as monokine induced by gamma interferon (MIG) (18161± 6405 pg/ml), compared to healthy controls or pediatric patients with sJIA during active disease. Two whole blood RNA samples collected from our Candle patient were compared to 6 whole blood RNA samples collected from healthy controls comparable for age. We obtained results consistent with those obtained by serum cytokine measurement: IFNg, CXCL10 and CXCL11 mRNA expression were significantly increased compared to healthy controls (1.88, 32.7 and 4.1 fold higher respectively). We also found that the expression of the HLA-DRB1, a gene whose expression is known to be induced by IFN-gamma, was markedly upregulated (> 100.000 fold increase) compared to healthy controls.

## Conclusion

In conclusion, we describe a typical CANDLE clinical phenotype in the absence of biallelic mutations of PSMB8 gene. The presence of high levels of IFN-γ and of IFN-γ related chemokines points to a major pathogenic role of the IFN-γ pathway, which appears to be similar to what has been recently reported in CANDLE (Liu et al). The pathogenic role of the variant T74S remains to be elucidated: a potential role is suggested by its presence in a patient with a classical phenotype and with a dysregulation of the IFN pathway, taking also into account that this variant is close to the know pathogenic mutation T75M.

## Disclosure of interest

None declared.

